# Pandemic (H1N1) 2009 in Breeding Turkeys, Valparaiso, Chile

**DOI:** 10.3201/eid1604.091402

**Published:** 2010-04

**Authors:** Christian Mathieu, Valentina Moreno, Patricio Retamal, Alvaro Gonzalez, Alejandro Rivera, Jorge Fuller, Cecilia Jara, Claudio Lecocq, Miriam Rojas, Alfonso García, Marcela Vasquez, Michel Agredo, Cristian Gutiérrez, Hector Escobar, Rodrigo Fasce, Judith Mora, Julio García, Jorge Fernández, Claudio Ternicier, Patricia Avalos

**Affiliations:** Servicio Agrícola y Ganadero, Santiago, Chile (C. Mathieu, V. Moreno, A. Gonzalez, A. Rivera, J. Fuller, C. Jara, C. Lecocq, M. Rojas, A. García, M. Vasquez, M. Agredo, C. Gutiérrez, H. Escobar, C. Ternicier, P. Avalos); Universidad de Chile, Santiago (P. Retamal); Instituto de Salud Pública, Santiago (R. Fasce, J. Mora, J. García, J. Fernández)

**Keywords:** Influenza, pandemic (H1N1) 2009, turkeys, viruses, Chile, dispatch

## Abstract

Pandemic (H1N1) 2009 virus was detected in breeding turkeys on 2 farms in Valparaiso, Chile. Infection was associated with measurable declines in egg production and shell quality. Although the source of infection is not yet known, the outbreak was controlled, and the virus was eliminated from the birds.

Influenza A pandemic (H1N1) 2009 virus is a novel highly transmissible agent that contains a unique combination of gene segments from different swine lineages (*1*); it has circulated in humans since April 2009 (*2*). First detected in North America, the virus was disseminated worldwide in just a few weeks, prompting the World Health Organization to raise its global health alert to the pandemic stage (*2**,**3*).

By December 2009, a total of 14 countries had reported that the pandemic strain was infecting swine, generating concern about the role of other susceptible species in the viral epidemiology. Fortunately, only a mild respiratory disease developed in the ill swine, and outbreaks were controlled with biosafety measures, avoiding dissemination to humans and animals (*4*). In Chile, the first case of human infection with the pandemic strain was confirmed on May 17, 2009; infection increased within 6 months to a total of 12,276 cases, with 147 deaths (*5*).

On July 23, 2009, in the Valparaiso Region of Chile, 1 flock (A1) from a commercial turkey breeding farm (farm A) started to show a measurable decrease in egg production and shell quality (Figure 1). During the following 2 weeks, similar signs were observed in 3 other flocks (A2, A3, A4) at farm A and 2 flocks (B1 and B4) on another turkey breeding farm (farm B) 50 km away, both belonging to the same company. However, neither respiratory signs nor increased death rates were observed. Because an influenza virus was suspected, on August 13 the situation was reported to the Chilean Agricultural and Livestock Service (SAG) for diagnosis.

**Figure 1:**
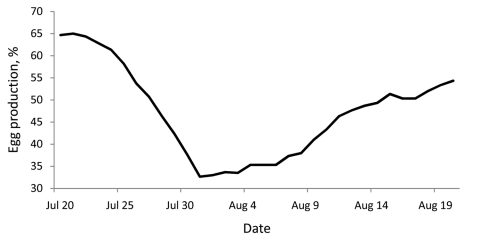
Average egg production of 3 pandemic (H1N1) 2009–infected turkey flocks (A1, A2, and A3) during July 20–August 20, 2009, Valparaiso, Chile. Production was calculated as a daily egg-laying rate (%).

## The Study

On August 14, the first blood samples were taken from turkeys of all affected flocks and submitted for serodiagnosis (Table). The agar gel immunodiffusion assay (AGID) detected antibodies to influenza A virus in 140 (62%) of 227 turkeys sampled. Farm A had a higher proportion of positives (80%) than farm B (32%) (odds ratio [OR] 8.4; 95% confidence interval [CI] 4.6–15.5).

**Table:** Laboratory results of testing conducted on turkey farms affected by the pandemic (H1N1) 2009 influenza outbreak, Valparaiso Region, Chile, 2009*

Farm	Flock no.				Results					
		Turkeys			AGID			rRT-PCR		
		No.	Age, wk		No. animals sampled	No. positive		No. animals sampled	No. positive	Ct
A	1	7,032	53		28	18		26	3	31.4
	2	4,410	77		28	25		43	1	34.8
	3	7,556	49		28	17		19	0	
	4	6,922	37		56	52		42	0	
B	1	7,004	45		28	2		30	3	34.9
	4	5,950	57		59	26		47	6	27.1

*AGID, agar gel immunodiffusion assay; rRT-PCR, real-time reverse transcription–PCR to detect matrix protein; Ct, cycle threshold values of positive results (averages).

Because of this finding, SAG adopted several control measures. Involved premises were quarantined and biosecurity standards were intensified, an epidemiologic investigation of the outbreak was initiated, and postmortem examinations of some carcasses were conducted. Each farm was considered an epidemiologic unit because each contained several flocks located closer than 1,000 m inside the same farm and were managed under independent biosecurity measures. On August 16, sampling for viral RNA detection by real-time reverse transcription–PCR (rRT-PCR) (*6*) was conducted, including not only affected flocks but surrounding premises; cloacal and tracheal swabs and embryonated eggs were collected from hatcheries. In addition, neighboring turkey farms were sampled for serologic analysis.

By August 18, results of this screening showed infection only in breeder turkeys from the first affected flocks of farms A and B. The rRT-PCR identified RNA corresponding to the influenza A matrix gene (Table) but not RNA of H5 or H7 genes. In some cloacal and tracheal swabs, viral RNA was detected, with cycle threshold values of 22.3–36.0. In the homogenates of embryo lungs and tracheas, viral RNA was not detected, suggesting that no vertical transmission occurred in this outbreak. Necropsies of 3 birds showed salpingitis, peritonitis, and interruption of follicular development. No other lesions were observed, and birds that had symptoms at the beginning of the outbreak were recovering and returning to normal laying rates after 3 weeks (Figure 1). Pools of feces belonging to 12 wild birds, including ducks (*Anas georgica*) and coots (*Fulica armillata*), collected near farm A were negative for influenza virus by rRT-PCR.

In addition, the SAG laboratory had been working on subtyping the agent by developing hemagglutinin and neuraminidase inhibition tests (*7*) using 5 AGID-positive serum samples. Subtype H1N1 was found after all 15 hemagglutinin and 9 neuraminidase subtypes were tested.

On August 19, SAG authorities coordinated with the Chilean Public Health Institute (ISP) for the virus isolation by using specific pathogen-free chicken embryos and MDCK cells (*8*) and for sequencing the viral genome (*9*) because the diagnosis for the pandemic agent was centered in the ISP facilities. In addition, an RNA sample was sent to the National Veterinary Services Laboratories (US Department of Agriculture), the World Organization for Animal Health reference laboratory for avian influenza.

On August 27, the viral sequences had been informed by ISP and National Veterinary Services Laboratories (GenBank accession nos. GQ866230, GQ866231, GQ866229, GQ866225, GQ866227, GQ866226, GQ866232, and GQ866228), having an almost complete identity with the novel pandemic strain. These results suggested transmission of pandemic (H1N1) 2009 virus from humans to birds.

**Figure 2:**
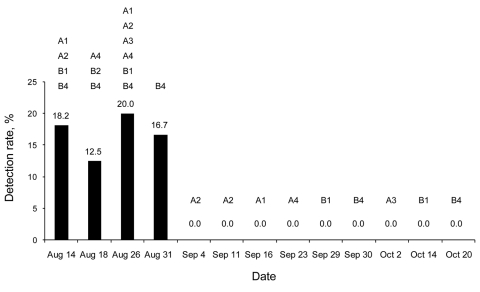
Pandemic (H1N1) 2009 virus detection rates (%) in affected turkey flocks from farms A and B during August 14–October 20, 2009, Valparaiso, Chile. Tracheal and cloacal swabs were analyzed by real-time reverse transcription–PCR to detect matrix and N1 genes. In each sampling date, detection rates appear in numbers, and sampled flocks are indicated by letters and numbers.

In the follow-up to this outbreak during September and October, SAG implemented an rRT-PCR assay developed by Southeast Poultry Research Laboratory (Athens, GA, USA) for the detection of the pandemic (H1N1) 2009 N1 gene (*10*). When egg production began progressively recovering in flocks, cloacal and tracheal swabs were analyzed for viral detection by using rRT-PCR assays. The last evidence of infection was obtained on August 31 (Figure 2), suggesting that the virus was eliminated from turkeys after 2–4 weeks.

## Conclusions

The source of infection for animals is still under investigation, but no clinical signs consistent with influenza-like illness were reported in staff working on the farms. Viral dissemination might have occurred either from the same infectious source, i.e., a farm worker or through fomites transported between premises. In any case, the biosafety system implemented in the company was working mainly to prevent infections from wild animals but not from humans. Transmission among animals was expected to have occurred through direct contact with secretions and feces from infected to susceptible birds.

The low cycle threshold values found by rRT-PCR in some samples suggest that the virus can replicate in turkeys, although previous attempts to experimentally infect turkeys with this strain had been unsuccessful (*10*). Some unknown predisposing factor expressed in the farm environment has transformed these birds into reservoirs of the pandemic strain. This event, also detected in Canada and the United States (*4*), could be explained by the reported presence of both avian and mammalian influenza A receptors in tissues from turkeys. Indeed, these turkeys are susceptible to a wide variety of influenza A viruses, including those from wild birds and swine, which are detected in these birds frequently (*11*). The role of turkeys in the epidemiology of pandemic (H1N1) 2009 strain remains to be elucidated.

In this outbreak, the infection in turkeys was mild, with reproductive symptoms and natural recovery. Some authors have reported a similar situation when these birds were experimentally infected with the triple reassortant H3N2 subtype isolated from pigs (*11**,**12*). Because some segments of subtype H3N2 are in the lineage of pandemic (H1N1) 2009 virus (*2*), the genetic bases for the interspecies transmission of this strain might be found on these segments. The turkeys involved in the outbreak were held under strict biosafety measures and sent to slaughter after laboratory verification of viral disappearance. The control strategy promptly and completely eliminated the risk to humans and animals.
